# Experience-dependent neurodevelopment of self-regulation in adolescence

**DOI:** 10.1016/j.dcn.2024.101356

**Published:** 2024-02-09

**Authors:** Wesley J. Meredith, Jennifer A. Silvers

**Affiliations:** Department of Psychology, University of California, Los Angeles, 1285 Franz Hall, Los Angeles, CA, USA

**Keywords:** Social context, Structural environment, Training, Brain development, Puberty, Adolescence

## Abstract

Adolescence is a period of rapid biobehavioral change, characterized in part by increased neural maturation and sensitivity to one’s environment. In this review, we aim to demonstrate that self-regulation skills are tuned by adolescents’ social, cultural, and socioeconomic contexts. We discuss adjacent literatures that demonstrate the importance of experience-dependent learning for adolescent development: environmental contextual influences and training paradigms that aim to improve regulation skills. We first highlight changes in prominent limbic and cortical regions—like the amygdala and medial prefrontal cortex—as well as structural and functional connectivity between these areas that are associated with adolescents’ regulation skills. Next, we consider how puberty, the hallmark developmental milestone in adolescence, helps instantiate these biobehavioral adaptations. We then survey the existing literature demonstrating the ways in which cultural, socioeconomic, and interpersonal contexts drive behavioral and neural adaptation for self-regulation. Finally, we highlight promising results from regulation training paradigms that suggest training may be especially efficacious for adolescent samples. In our conclusion, we highlight some exciting frontiers in human self-regulation research as well as recommendations for improving the methodological implementation of developmental neuroimaging studies and training paradigms.

## 1 Introduction

Adolescence is a formative period for environmental input across multiple biobehavioral systems due to heightened plasticity. Greater structural and functional brain plasticity, for example, drives neural and psychological adaptation as adolescents navigate their expanding and increasingly complex social and cognitive environments. In humans, however, it is difficult to directly measure neural plasticity due to the invasiveness of methods capable of observing single-neuron and circuit-level processes. Instead, as discussed in Section 1.2 , our understanding of human adolescent plasticity comes from indirect measures of structural and functional changes in brain maturation (e.g., reductions in gray matter volume, greater spatial focalization of neural activity; Berl et al., 2006; Selemon, 2013; Zatorre et al., 2012). Our understanding of the cellular and molecular processes undergirding adolescent plasticity are further supported by non-human research, which shows that over-expression of receptor sites and heightened activity for neurotransmitters (e.g., GABA, dopamine, serotonin) during this period coincides with waves of distributed synaptic pruning across regions and synaptic growth from the amygdala to cortical regions (for reviews, see Larsen and Luna, 2018; Sturman and Moghaddam, 2011)**.**

Concurrently, physiological changes can alter the way adolescents understand and interact with their environments, and how adolescents are perceived within their social contexts. Self-regulation, the collection of skills that serve to regulate behavior, thoughts, and emotions in goal-congruent ways (Gestsdottir and Lerner, 2008, Luna et al., 2015, Schweizer et al., 2020), is crucial for promoting wellbeing during this period of neural and physiological change. The current review aims to unpack how contexts and experiences tune neurodevelopment during adolescence, with an express focus on self-regulation, as these skills appear to develop considerably during this period. We characterize experience-dependent self-regulation development in three main parts. First, we survey general trends in behavioral and neural development subserving self-regulation, with a particular focus on emotion regulation. We build on this foundation by describing mechanisms through which biobehavioral plasticity facilitates neural adaptation in emotion and cognition-related brain regions. For the second part of our review, we enumerate how environmental contexts—both structural and social—can promote adaptive and maladaptive self-regulation outcomes in adolescents. We consider aspects of cultural, socioeconomic, and interpersonal contexts as important features of adolescents’ environments that help drive neural adaptation. Additionally, we consider how variations in pubertal timing may interact with environmental features to impact emotion development and psychopathology. In the final part of our review, we highlight self-regulation training paradigms as an experimental means of modifying experience. Here, we consider how frequent and varied environmental exposures can leverage experience-dependent neural adaptation during adolescence to nourish and promote healthy emotion regulation skills in adolescence. In our concluding remarks, we synthesize the key takeaways from each portion of our review to set forth recommendations for future inquiry.

### 1.1 Defining adolescence

Adolescence is a period of biopsychosocial change traditionally defined as beginning around puberty and ending when individuals achieve “independence” (Busso et al., 2018). Given these somewhat vague markers, it is perhaps unsurprising that studies often employ variable criteria when conceptualizing adolescence (Sawyer et al., 2012, Sawyer et al., 2018). Consistent with what is most commonly observed in the developmental cognitive neuroscience literature, the present review primarily focuses on studies including individuals between ages 10 and 25 years as evidence for adolescent neurodevelopment. We offer additional discussion on the variability in conceptualizing adolescence in Section 4 .

### 1.2 Developmental changes in self-regulation during adolescence

Self-regulation involves regulating behaviors, cognitions and emotions in accordance with one’s goals (Gestsdottir and Lerner, 2008, Luna et al., 2015, Schweizer et al., 2020). Adolescence presents as a time of considerable change with regards to the neural and behavioral bases of self-regulation. Prior work has demonstrated that the ability to regulate cognition and cognition-related actions—often referred to as executive functions or cognitive control—change appreciably during childhood and early adolescence before approaching adult-like performance in middle to late adolescence (Diamond, 2013, Kim-Spoon et al., 2021). Critically, however, different developmental timescales have been observed for different constituent components of executive function as well as for self-regulation occurring in different contexts. For example, performance appears to continue improving with age in the 14–18 year range for inhibitory control tasks, but not with working memory or task switching (Theodoraki et al., 2020). Furthermore, even within the inhibitory control domain, different maturational trajectories may emerge based on how performance is operationalized. For tasks involving motor inhibition (e.g., Flanker, anti-saccade), performance is generally adult-like by middle or later adolescence (Diamond, 2013, Klein and Foerster, 2001), whereas youth may continue showing age-related improvements on non-motor tasks (e.g., verbal Stroop; Theodoraki et al., 2020) or self-report measures (Atherton et al., 2020) of cognitive control domains into later adolescence and emerging adulthood. Additional variability in adolescent inhibitory performance is introduced when manipulating motivational contexts (e.g., monetary or social incentives; Bowers et al., 2021; Sharp et al., 2022), inhibition types (e.g., proactive vs. reactive control; Andrews-Hanna et al., 2011), or varying rule sets (Velanova et al., 2008, Velanova et al., 2009). Taken together, these findings suggest that the building blocks of motor inhibitory control are set by mid-adolescence but the ability to consistently deploy inhibitory control in more cognitively demanding or complex settings continues to develop into emerging adulthood, as do self-regulatory skills associated with other features of cognitive control.

The ability to self-regulate emotions presents on a more protracted timeline than non-emotional self-regulation across adolescence and emerging adulthood (McRae et al., 2012, Silvers et al., 2012). Emotion regulation—the ability to adaptively modify internal emotional states and external emotional reactions in accordance with one’s goals (Gross, 1998)—is a core competency that sees rapid developmental change during adolescence. Given the particularly pronounced changes surrounding emotion regulation during adolescence, we focus on its developmental features as an exemplar case for understanding the development of self-regulation.

Emotion regulation can be used to maintain, increase, or decrease responses to and behaviors surrounding both reward and threat. The ability to regulate reward- and threat-related emotions is particularly important during adolescence because adolescents demonstrate heightened reward sensitivity in the ventral striatum (VS) compared to children and adults (Braams et al., 2015, Galván et al., 2006, Silverman et al., 2015, van Duijvenvoorde et al., 2016), as well as heightened threat sensitivity relative to adults (Dreyfuss et al., 2014, Spielberg et al., 2014, Spielberg et al., 2015). These propensities are important for encouraging adolescents to seek out a greater range of social and affective experiences, and this in turn may promote experience-dependent emotion learning (Gee et al., 2018). Specifically, exposure to a range of affective experiences provides opportunities for practicing and improving emotion regulation skills. Consistent with this framing, several studies have demonstrated age-related improvements in deployment of top-down regulation of emotion during the adolescent period (Gee et al., 2013, Hare et al., 2008, Silvers, 2020).

During adolescence, regulatory skills become more complex and self-directed (i.e., less caregiver-dependent) as youth develop their capacity to engage in emotion regulation and to better match situation-appropriate skills for a given context. For example, cognitive reappraisal is a hallmark regulation strategy that modulates emotion by updating the user’s personal or narrative connection to a stimulus as a means to change the emotional import (John and Gross, 2004). Youth begin to utilize reappraisal strategies more successfully in late childhood, and these skills linearly improve into adolescence before reaching adult-like performance in later adolescence and emerging adulthood – following a similar, but somewhat more protracted trajectory to what is commonly observed in non-affective cognitive control skills like inhibitory control (Diamond, 2013, Kim-Spoon et al., 2021, McRae et al., 2012, Silvers et al., 2012). While emotion regulation skills improve during adolescence for most individuals, many emotion-related psychopathologies also emerge or worsen during this period (Lee et al., 2014), which may in part be driven by maladaptive emotion regulation development and related cognitive control skills (Gabrys et al., 2018, LaMontagne et al., 2022, Simonds et al., 2007). In particular, emotion-related psychopathologies during adolescence may reflect suboptimal context-strategy pairing across situations or difficulty in successfully engaging a particular regulatory skill (e.g., failure to extinguish negative affect for a familiar, non-threatening stimulus; Compas et al., 2017; McLaughlin et al., 2011; Silk et al., 2003). This comports with some work showing adolescents may rely on regulation strategies like withdrawal or rumination that are considered maladaptive (Cracco et al., 2017), while also utilizing a smaller repertoire of strategies across emotional contexts (Zimmermann and Iwanski, 2014). Considering the developmental trajectories of emotion regulation development alongside the risk for psychopathologies, adolescence appears a promising intervention window for nourishing healthy experience-dependent emotion regulation development (Silvers, 2020, Silvers, 2022).

### 1.3 Structural and functional mechanisms of adolescent neural adaptation

During adolescence, experience-dependent self-regulatory development unfolds through protracted structural maturation and changing functional connections within- and between cortical and subcortical areas. Structural brain characteristics—like cortical thickness, surface area, and volume—develop dynamically along region-specific linear and non-linear trajectories (Juraska and Willing, 2017, Tamnes et al., 2010, Tamnes et al., 2017). Subcortical regions supporting motivation and reward like the amygdala and nucleus accumbens mature earlier compared to dorsal and lateral regions of prefrontal cortex (PFC) that support cognitive control (Casey et al., 2016, Casey et al., 2008a, Casey et al., 2008b, Mills et al., 2014, Somerville et al., 2010). Structural maturation of brain regions related to social cognition, including temporal parietal junction (TPJ) and medial prefrontal cortex (mPFC) also show linear and nonlinear developmental trajectories for gray matter volume, surface area, and cortical thickness throughout later childhood and adolescence (Mills et al., 2014). Importantly, brain areas that support social cognition tend to mature in accordance with the growing influence of peers in adolescents’ lives (Gardner and Steinberg, 2005, Helsen et al., 2000), suggesting a link between changing social contexts and the neurodevelopment needed to navigate such changes.

Distributed networks across the brain subserving self-regulation also undergo considerable maturation during adolescence. For example, regional and whole-network changes are observed in prefrontal areas like mPFC, ACC, and striatum that undergird core cognitive control and emotion regulation skills (Botvinick and Braver, 2015, Dwyer et al., 2014, Luna et al., 2013). At the same time, age is associated with less amygdala reactivity and a ventral-to-dorsal shift in prefrontal recruitment during emotional processing in adolescence (Silvers et al., 2017), marking a shift toward greater top-down control of affective processes in adolescents – perhaps in part due to their accumulated experiences with managing emotion. Corticolimbic connections between the amygdala and prefrontal regions like mPFC also undergo sustained pruning across adolescence into early adulthood, with particularly prominent changes occurring in the strength and directionality of functional connectivity that enable greater top-down control (Ahmed et al., 2015, Gee et al., 2013, Gee et al., 2022, Saygin et al., 2015). Experience guides brain connectivity by alternatively strengthening and weakening network connections in accordance with the demands of one’s environment. For example, functional connectivity between the amygdala and mPFC in youth longitudinally predicts resting state connectivity between these regions over a two-year period (Gabard-Durnam et al., 2016), demonstrating that phasic environmental exposures drive long-term adaptation in adolescent network organization. Moreover, network characteristics (e.g., modularity) differentially relate to emotion regulation skills across common networks, with greater modularity in cognitive control networks but less modularity in default mode networks both relating to improved adolescent regulation skills, suggesting that experience with emotion regulation hones networks to promote better skills (Guassi Moreira et al., 2021). Together, regional and network structural development promote greater effortful regulation of environmental cues and their impact on internal states.

Variation in environmental contexts ostensibly reinforces experience-dependent optimalization of regulatory strategies by offering adolescents the opportunity to strengthen their strategy selection proficiencies and adapt behavioral and cognitive processes underlying such strategies (Pesce et al., 2019). Put another way, synergistic coupling across cognitive, behavioral, and neural regulatory processes entrains context-dependent regulatory patterns that can be outfitted to novel contexts (Yang et al., 2019). From a specialization framework, this biobehavioral synergism could help entrain context-dependent tuning of neurons and networks that support specific regulatory brain states. Prior research suggests experience-dependent neurodevelopment is instantiated through neural focalization in regions of the brain subserving self-regulatory functions like inhibitory control (Durston et al., 2006) and mentalizing (Johnson et al., 2009). Similar results have been observed in the context of emotion regulation, such that adolescents who demonstrate greater neural focalization in ventrolateral PFC (vlPFC) report less negative affect when regulating responses to negative emotional stimuli (Guassi Moreira et al., 2019). This developmental shift toward focalized activity within brain regions may support self-regulation neurodevelopment by fortifying patterns of local and distributed brain activity that can be flexibly engaged or updated in novel contexts. However, sustained exposure to adverse or limited environments may instead reinforce or entrain suboptimal or dysregulated patterns of regulatory activity (McLaughlin et al., 2019).

## 2 Environmental experiences driving self-regulation development

Prior research suggests that concurrent neural maturation and changing social and environmental contexts drive self-regulation neurodevelopment during adolescence. Experiences in adolescents’ homes, schools, and neighborhoods—either measured directly or through indirect constructs like socioeconomic status (SES)—play a key role in shaping self-regulation neurodevelopment. For example, greater support at home and school positively relates to indicators of greater structural brain maturation (e.g., cortical myelination, cortical thinning), whereas greater family conflict instead relates to features of less mature brain macrostructure (e.g., greater cortical thickness; Hong et al., 2021). Adolescents’ experiences provide opportunities to develop and hone self-regulation skills across multiple contexts. These experiences both shape brain development (i.e., experience-dependent development), and are shaped by brain development (e.g., prefrontal development enables greater execution of self-regulatory strategies). In this section, we survey literature that highlights how variations in cultural, socioeconomic, interpersonal, and pubertal contexts relate to adolescent self-regulation neurodevelopment. Importantly, in some instances we describe changes in brain or behavior that are often construed as maladaptive based on associated outcomes in adolescence and beyond (e.g., internalizing symptoms). However, it is important to also acknowledge there is no singular path for optimal development, and developmental trajectories likely vary in accordance with an individual’s context. For example, both within- and between-individuals, using strategies commonly labeled “maladaptive” (e.g., disengagement, hypervigilance) may actually be adaptive and appropriate for certain contexts (e.g., environments with high emotional intensity or greater unpredictability; De France and Evans, 2021; Holmes et al., 2019; Phan et al., 2020; Wenzel et al., 2022).

### 2.1 Cultural contexts

Seminal cross-cultural work suggests that caregivers model regulatory behavior in line with cultural values (Camras et al., 2014, Halberstadt and Lozada, 2011). The developmental implications of socializing different regulatory behaviors also appear contextually dependent on the cultural appropriateness of those skills. For example, using emotion regulatory strategies like expressive suppression—or, not showing what one is feeling—is generally construed as maladaptive in Western circles, but appears unrelated to measures of negative affect, like depressive symptoms, in other cultures that value collectivism (Matsumoto et al., 2008a, Matsumoto et al., 2008b, Soto et al., 2011). Overlaps between cultural and ethnic identities, discrimination, and oppression can also influence how caregivers socialize self-regulatory strategies and how their children develop within their broader sociopolitical environments. For example, Black caregivers—who disproportionately experience racially targeted oppression and state violence in the United States (Duncan et al., 2014; McLoyd, 1990; Sheats et al., 2018)— may teach their children to be vigilant toward threats of racism and violence, while simultaneously socializing flexible regulatory strategies for managing emotional responses to threat (Dunbar et al., 2015, Dunbar et al., 2017, Lozada et al., 2022).

While influential theoretical frameworks have suggested that adolescence is a sensitive developmental period for cultural influences on adolescent neurodevelopment in general (Blakemore and Mills, 2014, Choudhury, 2010), and on self-regulation specifically, little work has investigated how culture shapes self-regulation neurodevelopment in adolescence. Most of what is known about how culture shapes cognitive and affective brain function comes from work in adults. For example, meta-analytic evidence suggests that individuals from Western, individualistic cultures more strongly recruit regions correlated with self-reflection and emotional evaluation (e.g., vmPFC, ACC) when viewing emotional stimuli while individuals from Eastern, collectivist cultures instead recruit regions related to theory of mind and self-regulation (e.g., dorsal mPFC, TPJ, and lateral PFC; Han and Ma, 2014). Such findings suggest that socialization practices tune the brain to adopt more self- or other-oriented perspectives during emotional processing, in accordance with cultural norms. Initial work further suggests that cultural norms may shape key features of adolescent brain function, including reward sensitivity. Latine adolescents, for example, have shown greater reward-related striatal and ventral tegmental activity when winning money for their family at their own expense compared to white adolescents (Telzer et al., 2010). More recently, another study provided preliminary evidence that culture may also shape neural function during social interactions. Specifically, Rapp et al. (2022) observed that cultural ideals can moderate relationships between caregiver socialization and neural function during interpersonal exchanges, such that adolescents who endorsed more collectivistic ideals showed weaker electroencephalographic signatures of aversion (feedback-related negativity) to negative peer feedback during a task compared to participants with fewer endorsements of collectivistic ideals. Given these findings on how culture shapes reward and socioemotional function, it stands to reason that culture may also impact adolescent neurodevelopment related to self-regulation. It would be valuable for future work to test whether culturally dependent regulation strategies relate to observable differences in top-down regulatory circuit maturation, and whether such circuit differences also relate to variation in regulatory performance across contexts.

### 2.2 Socioeconomic contexts in the home and neighborhood

In this section, we highlight the ways material and structural environments relate to neurodevelopmental trajectories of adolescent self-regulation skills with a particular focus on socioeconomic status (SES). SES is a construct that approximates resources and possibility at youth’s disposal, rather than a deterministic trait, commonly derived using measures of caregiver occupational prestige, income, and educational attainment (Mueller and Parcel, 1981, Sirin, 2005). For youth, SES is correlated with cognitive and socioemotional development and academic achievement (Bradley and Corwyn, 2002, Noble et al., 2007; White, 1982). These relationships are likely in part driven by SES providing varied contexts for experience-dependent neurodevelopment, which subsequently yield different neurodevelopmental outcomes. SES has been shown to longitudinally track with global brain structure maturation (e.g., cortical thickness) across development fairly consistently, such that socioeconomic disadvantage by and large tracks with attenuated structural growth trajectories from infancy through adolescence (McDermott et al., 2019, Rakesh et al., 2023). That said, the impact of these adaptations may manifest differently across developmental stages depending on which specific features of brain development are undergoing the most marked changes when experiences occur. In adolescence specifically, greater socioeconomic advantage is most strongly associated with global measures of brain morphology (e.g., volume, surface area, cortical thickness) in frontal and subcortical regions associated with emotion regulation and cognitive control, like lateral PFC, ACC, temporal and superior parietal regions, and the amygdala (Buckley et al., 2019, Luby et al., 2013, McDermott et al., 2019, Noble et al., 2015, Rakesh and Whittle, 2021). This suggests that as cognitive learning unfolds during adolescence, maturing corticolimbic circuits and prefrontal regions may be especially receptive to input from environmental features proximally measured by SES, including reliable access to supportive learning environments inside and outside the home. Moreover, recent work has not only made connections between SES and cognitively stimulating home environments, but has also shown that the cognitive stimulation afforded by socioeconomic advantage may also help explain observed relationships between greater white matter microstructural maturation (e.g., increased fractional anisotropy), enhanced frontoparietal neural recruitment during working memory tasks, and academic achievement in adolescents (Rosen et al., 2018). Rakesh and Whittle (2021) also note that composite measures of SES—as opposed to unidimensional measures like income—bear the most consistent associations with brain morphology across the literature, suggesting that multivariate approaches best capture the multifaceted social and structural features encapsulated by this construct and are thus better equipped to leverage the diverse features of SES to relate with neural and behavioral outcomes. Together, these findings suggest that features of SES likely contribute to the structural and functional maturation of corticolimbic regions related to emotion processing and self-regulation, including regions of prefrontal cortex and the amygdala. In particular, greater socioeconomic advantage likely helps promote experience-dependent neural adaptation by enabling access to supportive learning environments.

SES may additionally impact regulatory control of goal-directed behaviors, especially in relation to depressive symptoms and neural functioning. Adolescents from lower SES backgrounds show lower ACC activity and broader (i.e., less mature) recruitment of medial frontal regions during cognitive tasks alongside greater depressive symptoms (Brieant et al., 2021, Palacios-Barrios et al., 2021, Rudolph et al., 2014), suggesting that resource deprivation may blunt processes that drive goal-relevant behavior, including successful assignment of positive reward values to certain kinds of stimuli. SES tends to be highly correlated with other environmental experiences that must be teased apart when identifying experiential influences on brain development. For example, adolescents growing up in lower SES environments are more likely to encounter neighborhood violence, which in turn appears to impact affective processing and self-regulatory behaviors (Markowitz, 2003). Violence-exposed youth preferentially attend to negative stimulus features, more frequently engage in emotional suppression, and show altered corticolimbic connectivity alongside heightened amygdala reactivity in response to threats (Hyde et al., 2022, McCoy et al., 2016, Suarez et al., 2022; White et al., 2019). When considered through an adaptive lens, this suggests that self-regulatory architecture develops in accordance with one’s environmental demands—specifically, that more threatening contexts are more likely to yield vigilant brains.

### 2.3 Close interpersonal contexts

#### 2.3.1 Caregiver influences on adolescent self-regulation development

Caregivers are powerful conduits and generators of social contexts for their children across development, and the mere presence of caregivers can shape early brain and behavioral development (Jessen, 2020, Moriceau and Sullivan, 2006). Early in their children’s lives, caregivers begin modeling regulatory behaviors (Morris et al., 2007, Morris et al., 2011, Morris et al., 2017), and these socialization practices predict greater down-regulation of amygdala reactivity in later adolescence, marked by less positive amygdala-vmPFC functional connectivity while viewing negative emotional stimuli (Chen et al., 2020). Other work also suggests that negative caregiving experiences like exposure to maltreatment or parental conflict are linked to psychopathologies and augmented amygdala-PFC connectivity—which may serve as a potential protective factor against internalizing symptoms (Herringa et al., 2016, Morris et al., 2007, Raposo and Francisco, 2022, Weissman et al., 2019). However, this enhanced amygdala-PFC coupling was not observed in adolescents with greater internalizing symptoms, suggesting that higher doses of adversity may blunt compensatory mechanisms that help dampen amygdala hyperactivity to negative stimuli (Herringa et al., 2016). Notably, the timing of caregiving adversity is important when examining associations with amygdala-PFC connectivity, such that early caregiving adversity—experienced before adolescence, but not during adolescence—seems to most strongly predict later neural adaptations in amygdala-prefrontal circuits (Burghy et al., 2012, Herringa et al., 2013). Taken together, amygdala-PFC circuitry seems to be one candidate pathway through which early caregiving experiences entrain self-regulatory neural function in adolescence, either through promoting healthy development of amygdala function and amygdala-PFC connectivity, or by instead impeding compensatory top-down control of amygdala reactivity to negative stimuli at higher levels of caregiving adversity.

Far more research has been devoted to caregiver socialization in childhood, but emerging evidence highlights the continued role that caregivers play in socializing self-regulation skills for their adolescents. Familial cohesion and support seem to protect against adolescent internalizing problems, which likely reflects, among other things, healthy self-regulation skills (Raposo and Francisco, 2022). Caregiving adversity (e.g., abuse or neglect) can instead result in greater utilization of suboptimal regulation strategies like emotional suppression and rumination (Weissman et al., 2019). These regulative adaptations to adversity appear linked to higher emotional reactivity in adolescents and longitudinally predict psychopathologies across the lifespan (Weissman et al., 2019). Importantly, the relationships between familial adversity and regulatory neurodevelopment likely differentially manifest depending on developmental stage, where exposures in childhood shift attentional biases toward threat cues but instead shift attention away from threat in adolescence. Longitudinal outcomes based on interpersonal caregiving relationships have both phasic and tonic impacts on corticolimbic development. Phasic inputs from mere caregiver presence can help entrain top-down inhibitory regulatory activity by ramping vmPFC activity and promoting stronger amygdala-mPFC connectivity in adolescents (Rogers et al., 2020). Other phasic relationships attenuate by adolescence, such as the dampening of amygdala reactivity while viewing images of caregivers (Gee et al., 2014). Together, these findings suggest that caregivers evoke phasic effects over both cognitive and affective regulatory systems, although these effects manifest differently based on context and developmental stage. Maternal buffering of amygdala reactivity is apparent during childhood, while shifts toward greater self-directed regulation during adolescence coincide with the protracted functional maturation of top-down prefrontal circuits throughout adolescence and emerging adulthood. Tonically, caregiving behaviors like hostility are linked with stronger negative connectivity between the amygdala, prefrontal cortex, and ventral striatum—patterns suggestive of accelerated functional maturation—while viewing negatively valenced stimuli or engaging cognitive control (Gard et al., 2017, Kopala-Sibley et al., 2020). Similarly, caregiver deprivation (e.g., institutional orphanage care) is associated with more adult-like connectivity between prefrontal and subcortical structures like the amygdala and hippocampus (Gee et al., 2013, Silvers et al., 2016). These results suggest that negative caregiver socialization instantiates accelerated corticolimbic circuit maturation to enable self-directed regulatory capabilities in the absence of optimal caregiver co-regulatory support. Importantly, a recent review on caregiving behaviors and child neurodevelopment highlight methodological inconsistencies as a hindrance to our understanding of these complex biopsychosocial interactions (Farber et al., 2022). As research in this area accumulates, it will be imperative to consider ways of disentangling the intermingled familial social contexts influencing corticolimbic neurodevelopment as it relates to self-regulation.

#### 2.3.2 Peer influences on adolescent self-regulation development

During adolescence, shifting social environments make peer relationships increasingly impactful in shaping self-regulation. Relative to children, adolescents solicit social support from their peers more as they seek out support from caregivers and other adult figures less (Bokhorst et al., 2010, Spitz et al., 2020). Peers offer varied social referents and can help reorient regulation strategy selection in adolescents’ expanded social environments (Miller-Slough and Dunsmore, 2016), for example by modeling emotion regulation strategy usage (Christensen et al., 2022, Klimes-Dougan et al., 2014, Miller-Slough and Dunsmore, 2016, Reindl et al., 2016). While this can lead to shared suboptimal regulation strategies like co-rumination, the shared experiences within friend groups also appears to increase friendship quality (Rosen et al., 2007). Here, belonging to a network of peers—even a group with similar emotional difficulties (e.g., internalizing and externalizing behaviors; Miller-Slough and Dunsmore, 2016; Waldrip et al., 2008)—may be a contextually salient milestone for adolescents, and one that is valued as more important than optimally ameliorating emotional distress. Recent work in emerging adults suggests that peer support can impact in-the-moment emotional processing, where a friend’s reinterpretation of negative stimuli evokes less negative affect in individuals compared to reinterpreting negative stimuli on their own (Sahi et al., 2021). Notably, this suggests that even though maternal buffering effects may attenuate during childhood, shifts to peers as social referents during adolescence provides additional contexts through which co-regulatory behaviors are able to dampen negative affect. These results illustrate one way in which peers support one another, by scaffolding regulatory attempts. Considering the heightened importance of peers and friendships for adolescents, future work should examine whether adolescents might benefit from peer co-regulation more than younger children or adults (Sahi et al., 2023).

### 2.4 Puberty as a link between context and self-regulation development

Puberty is a biopsychosocial milestone that signals the onset of adolescence (Bello et al., 2017, Brooks-Gunn, 1984, Herbert et al., 2017). Increases in circulating gonadal hormones (estradiol, testosterone) during puberty play a prominent role in shaping brain organization and function, subsequently invigorating social motivational influences for adolescents (for comprehensive reviews, see Blakemore et al., 2010; Goddings et al., 2019). Variability in pubertal processes occurs naturally between individuals, however experiential factors can encourage earlier or later pubertal onset for some adolescents relative to their peers, which can subsequently affect how prominent self-regulation brain regions develop. For example, earlier pubertal onset in females^1^—and sometimes males—increases the risk for dampened emotional function in the amygdala (Forbes et al., 2011, Ladouceur, 2012, Whittle et al., 2015) and higher rates of internalizing and externalizing symptoms (Alloy et al., 2016, Ge et al., 2003, Ge and Natsuaki, 2009, Graber et al., 2004, Sontag et al., 2011). Below, we highlight a selection of environmental interactions with the onset of puberty, which in turn can influence adolescent neural and behavioral development.

#### 2.4.1 The influence of early life adversity on pubertal timing

Stress acceleration frameworks posit that experiences of early adversity may expedite neural maturation in order to meet environmental demands (Callaghan and Tottenham, 2016). Under such frameworks, earlier menarche may be one such mechanism that enables accelerated maturation in adolescence in response to their environments (Hamlat et al., 2023). Indirectly, early life adversity may also impact adolescent regulatory neurodevelopment by instantiating earlier pubertal development (Hamlat et al., 2021, Hamlat et al., 2022, Hamlat et al., 2023, Strong et al., 2016, Suglia et al., 2020; Zhang et al., 2019). Importantly, specific types of adverse experiences—for example, those that involve threat—are more strongly related to earlier menarche than those that do not (Hamlat et al., 2022). Moreover, connections between experiences and pubertal timing are not unidirectional. For example, earlier puberty also increases the risk for sexual abuse due to the earlier development of secondary sex characteristics (Herman-Giddens, 1988). This points to a bidirectional relationship between pubertal development and adolescent environments, whereby adverse experiences may initiate earlier pubertal maturation *and* earlier presentation of secondary sex characteristics may also subject adolescents to adverse experiences like abuse. In sum, the aforementioned evidence suggests that the reproductive system may respond to environmental demands both by changing its maturational timescale and, relatedly, by shaping hormone-dependent features of neurodevelopment.

#### 2.4.2 Racial and ethnic differences in pubertal timing

Larger proportions of non-Hispanic Black and Hispanic/Latine adolescents experience earlier pubertal onset compared to their non-Hispanic/Latine white peers (Anderson et al., 2003, Hamlat et al., 2022, Hamlat et al., 2023). Systemic socioeconomic disparities do not wholly explain earlier pubertal onset for racial and ethnic minorities relative to their white peers (Deardorff et al., 2014, Wu et al., 2002), and some work instead suggests Black adolescents from higher income families are more likely to experience earlier menarche (Braithwaite et al., 2009). At a minimum, socioeconomic advantage is not universally protective against early puberty for Black adolescents given the multiple ways systemic racism manifests across environmental contexts. Housing discrimination and environmental racism, for example, could also introduce broader exposure to environmental contaminants known to impact endocrine function and epigenetic adaptations (Lopez-Rodriguez et al., 2021, Osinubi et al., 2022). Racial discrimination could also be a prominent contributing stressor that impacts pubertal timing in Black adolescents (Shirazi and Rosinger, 2021), although more research is required to examine this association. However, earlier pubertal development may further expose Black adolescents to greater racial discrimination from their peers (Seaton and Carter, 2019) and exacerbate self-dysregulation and depressive symptoms (Gibbons et al., 2012, Woody et al., 2022). This means that transgenerational legacies and personal experiences of racism may not only impact pubertal timing for racial and ethnic minority adolescents, but also that earlier pubertal onset can increase the risk of personal experiences of racism and exacerbate stress-related adaptations for these youth. The bidirectional associations between earlier pubertal timing and experiences of systemic and interpersonal racism could either relate to accelerated maturation of top-down regulatory circuits or greater bottom-up amygdala reactivity (i.e., hypervigilance) depending on additional contextual inputs, but these speculative mechanisms require more longitudinal evidence from future work.

#### 2.4.3 Postponed pubertal development for some transgender youth

Adolescence can be a particularly stressful period for transgender youth, including non-binary and other genderqueer individuals, who may experience heightened dysphoria as their bodies develop physical traits associated with their assigned sex alongside greater victimization based on their gender presentation (Birkett et al., 2015, Connolly et al., 2016). Within the last two decades, puberty blockers have become more prevalent in the suite of gender affirming care options available to transgender adolescents (Hembree et al., 2017, Lopez et al., 2018). However, Lopez et al. (2018) additionally note racial and ethnic disparities in gender-affirming care practices, such that non-Hispanic white adolescents are more likely to receive puberty blockers as part of their care. While some have voiced concerns about inhibiting pubertal processes for transgender youth who would otherwise experience puberty “on-time” (Jorgensen et al., 2022), pubertal intervention can be life saving for transgender youth. Transgender youth receiving puberty blockers experience decreased suicidality alongside improvements to general affect and mental health (de Vries et al., 2011, Panagiotakopoulos et al., 2020, Rew et al., 2021). As transgender youth enter adulthood, hormone replacement therapies usher in a “second puberty,” which might help shift emotion-related activity in the amygdala and ACC toward healthy, adult patterns observed in their cisgender peers (Kiyar et al., 2022). The prospective benefits of using puberty blockers and other hormone therapies for gender affirming care seem promising, although more research is required to understand how postponed puberty might affect self-regulation neurodevelopment. Moreover, future research examining “second” pubertal development in transgender adults receiving hormone replacement therapies could offer novel insights and help disentangle puberty- and age-specific features of adolescent neurodevelopment.

## 3 Training emotion skills

Regulation training interventions complement the previously summarized findings linking environmental contexts and self-regulation development, as they create a quasi-experimental framework to examine more causal mechanisms through which experience impacts neurodevelopment. In this section, we examine evidence for the potential of emotion regulation training paradigms to promote and strengthen adaptive regulation strategies during adolescence. More work has targeted executive functioning and cognitive control skills as intervention targets in adolescent samples (e.g., Knoll et al., 2016; Lee et al., 2019; Zinke et al., 2012), so this section aims to highlight emotion regulation paradigms as a promising but understudied intervention. Though few studies have implemented emotion regulation training paradigms in healthy, typically developing adolescents, we present preliminary findings within this population alongside other research utilizing clinical and at-risk adolescent samples. Training paradigms implement varied methods, so this review additionally highlights key differences and takeaways garnered from the various ways these paradigms have been created for different target populations.

### 3.1 Initial findings surrounding training paradigms and adolescent emotional development

Training paradigms designed to improve healthy emotion regulation strategies appear successful for multiple populations across numerous developmental stages, suggesting that emotion regulation skills are targetable and modifiable. These paradigms build off the training success of executive function training paradigms that demonstrate consistent practice engaging cognitive skills can improve behavioral outcomes for multiple age groups —although the reach and transferability of these skills are limited by design constraints and age at intervention (Diamond and Ling, 2016, Karbach and Unger, 2014, Knoll et al., 2016; Lee et al., 2019; Thorell et al., 2009; Veloso et al., 2020; Zinke et al., 2012). Numerous studies have demonstrated that healthy adults who repeatedly practice using an emotional distancing strategy report sustained decreases in daily stress and negative affect (Denny et al., 2015, Denny and Ochsner, 2014, Ng and Diener, 2013). For children, training paradigms often incorporates dyadic exercises with a caregiver (Rothenberg et al., 2019), and these children have also shown lowered negative affect and problem behaviors. Given these promising results, emotion regulation training may be an especially effective intervention given the lower demands requested from participants, including time and resource investment, and the possibility for remote participation.

Fewer studies have implemented regulation training paradigms in healthy adolescent samples, however. In one sample of Iranian adolescent girls, training seemed to lower anxiety scores and curb suboptimal strategy usage (Nesayan et al., 2017). A much shorter, school-based training intervention reported immediate improvements in negative affect across four regulatory strategies (acceptance, distraction, reappraisal, problem solving), however these benefits did not persist at a one-month posttreatment follow up (Volkaert et al., 2020). Group regulation training for clinically at-risk adolescents appears to lower anxiety symptoms (Schuppert et al., 2009), although this study featured longer intervention sessions. Differences in implementation across all three studies, and the subsequent differences in training outcomes, point to a need for more robust replications and extensions of existing paradigms in order to find optimal features that bolster adolescents’ success.

Interventions indirectly targeting self-regulation, like mindfulness training, have yielded mixed results. Broadly, mindfulness encourages presence with one’s current physical and mental states and acceptance of one’s experiences (Bishop et al., 2004). For clinical samples, mindfulness is thought to buffer against adolescent psychopathologies by helping increase sensitivity toward one’s emotions and improving aspects of cognitive control (Broderick and Jennings, 2012, Sanger et al., 2018). However, a recent longitudinal study instead suggests that mindfulness can worsen some mental health symptoms over time, potentially making adolescents more aware of their negative emotions without providing tools to directly address them (Montero-Marin et al., 2022). This further illustrates the potential benefits in concretely training regulatory skills that will help adolescents dampen negative affect or increase positive affect.

Other regulation training paradigms have incorporated neurofeedback, a neuroimaging method that provides participants with real-time information on their functional brain activity as they attempt to change their brain state in pursuit of a neural or behavioral outcome (Watanabe et al., 2017). Preliminary work in healthy children and adolescents suggests youth can be trained to modulate emotion-related functional networks during a task by up-regulating insular activity or amygdala-prefrontal connectivity (Cohen Kadosh et al., 2016, Zich et al., 2020). In a clinical sample of adolescents, depressive symptoms appear partially ameliorated by up-regulating right amygdala function during an emotional task (Quevedo et al., 2020). In particular, it seems promising that a regional feedback target, like insular or right amygdala activation, could instantiate changes in up- and downstream information flow to other cognitive and affective brain regions, like the cingulate (Cohen Kadosh et al., 2016). However, neurofeedback paradigms are constrained by smaller samples and financial overhead that limits the feasibility of longitudinal follow ups. As these paradigms mature in the literature and potentially become more accessible using other imaging techniques (e.g., functional near infrared spectroscopy), future work should prioritize assessments of long-term success and adolescent-specific regional or network training targets that confer the most advantage to trainees.

These preliminary findings, combined with knowledge about self-regulation neurodevelopment and adolescent sensitive periods for experience-dependent learning (Fandakova and Hartley, 2020, Piekarski et al., 2017), suggest that adolescence may be an especially pivotal period to train regulation skills. Adolescents likely require frequent, consistent training experiences, as is suggested by conceptual training frameworks for adolescent executive functions (Berkman et al., 2012, Veloso et al., 2020). These paradigms may also offer insight into the ways neural plasticity supports cognitive learning through experience-dependent processes (Galván, 2010, Kadosh et al., 2013, Laube et al., 2020). In particular, exposure to variable regulation contexts or changes in task difficulty across sessions may bolster learning and transfer of optimal strategies to novel situations (Flegal et al., 2019). Relating behavioral and neural change following training, or targeting brain function in neural feedback paradigms, could help isolate the role of experience from ongoing experience-expectant development (Galván, 2010). Furthermore, online training success in younger children (Silk et al., 2020) and adolescents’ positive perceptions of remote, mobile-based clinical services (Lu et al., 2021), also suggest that online training modules could lower attrition rates and travel burdens while reaching broader samples of youth.

## 4 Summary and future directions

**Fig. 1:**
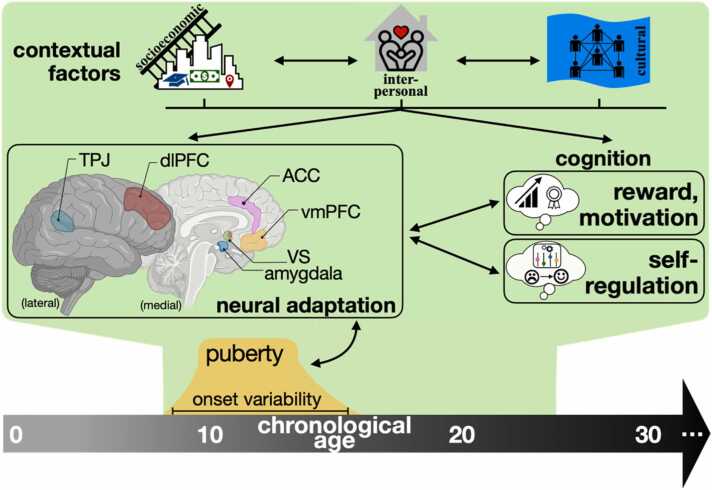
Conceptual diagram of adolescent neural adaptation and the influence of contextual factors on emotional neurodevelopment. Of special importance for affective learning and emotion regulation, corticolimbic connections are pruned over time while sensitivity to affective stimuli within specific prefrontal regions and the amygdala correspond with developmental stage. These changes promote adaptation as adolescents experience multiple contextual inputs from socioeconomic, interpersonal, and cultural influences. Double arrows reflect the capacity for forward- and backward-influence between contextual factors, brain adaptation and emotional skills. Figure created with inspiration from Ahmed et al. (2015) and Blakemore (2008). Brain graphic created with BioRender.com. In the **neural adaptation** portion, we highlight prominent regions discussed in relation to self-regulation development. From bottom right of **neural adaptation** brain figure (counterclockwise): amygdala, ventral striatum (VS), ventromedial prefrontal cortex (vmPFC), anterior cingulate cortex (ACC), dorsolateral prefrontal cortex (dlPFC), and temporal parietal junction (TPJ). Brief descriptions are provided below for each region. Amygdala: Multiple nuclei comprise this region, with connections to regions of vmPFC and dlPFC. The amygdala is associated with fear and threat responses as well as salience detection for affective stimuli. Ventral striatum (VS): A subcortical region interconnected with limbic regions (e.g., amygdala) and the prefrontal cortex. The ventral striatum is associated with evaluating reward and motivation for goal-oriented action (Silverman et al., 2015). The VS includes the nucleus accumbens, a portion adjacent to the septum that is implicated in reward processing through its involvement with action selection and integration of cognitive and affective information from distributed frontal and temporal regions (Floresco, 2015). Ventromedial prefrontal cortex (vmPFC): A subregion of the prefrontal cortex, vmPFC is a modulatory region, associated with extinguishing affective responses and engaging top-down systems for emotion responses and regulation (Buhle et al., 2014). Anterior cingulate cortex (ACC): A cortical region involved in executive function and emotion skills through extensive connections to regions of prefrontal cortex. The ACC is associated with goal maintenance and aspects of cognitive control (Gasquoine, 2013). Dorsolateral prefrontal cortex (dlPFC): Another subregion of the prefrontal cortex, the dlPFC is associated with aspects of attention and working memory. In relation to emotion regulation, it is thought to help maintain and update emotional information held in mind (Duncan and Owen, 2000). Temporal parietal junction (TPJ): Located along the border of temporal and parietal lobes, the temporal parietal junction serves as a nexus for multiple language, memory, and mentalizing. As such, the temporal parietal junction is a distinctly social brain region, providing social context to many behaviors, including emotionally laden social stimuli (Carter and Huettel, 2013).

Across multiple lines of research, evidence suggests that adolescence may be an important window for experiential influences on self-regulation development. During this period, neural maturation and pubertal processes drive changes in experiential learning and socially motivated behaviors. Meanwhile, adolescents navigate expanding social environments as they gain more autonomy, providing greater opportunity to incorporate inputs from multiple social others and adapt to changing environmental demands. In particular, caregivers and friends seem especially influential on the ways adolescents express and regulate their emotions. At the same time, cultural and socioeconomic contexts provide rich exposures to both positive and negative experiences that may promote neural adaptation undergirding cognitive control and emotion regulation development. Variability in pubertal timing and numerous puberty-related adaptations also appear to influence the way adolescents are perceived and how different environmental contexts may impart differential outcomes in emotion-related mental health. We also observe promising results in the efficacy of engaging adolescent emotion regulation in training paradigms, where these youth can hone skills over time through exposures to multiple exemplars where they might practice a particular skill or attempt to directly alter their acceptance (mindfulness) or brain state (neurofeedback) in-the-moment. Fig. 1 depicts the multiple pathways through which experience confers adaptation in adolescent development, namely through neural and cognitive outcomes as well as variability in pubertal processes. To conclude this review, below we outline three ways in which future neuroscientific work might better incorporate context into the study of self-regulation development during adolescence.

As we highlight throughout the review, adolescents’ culture, social relationships, socioeconomic background, and other environmental factors can impact self-regulation development. Thus, a critical avenue for future directions in this work is to sample diverse, representative, and community-based samples of adolescents to make stronger connections between the environment and experience-dependent neurodevelopment of self-regulation skills (e.g., The Adolescent Brain Cognitive Development Study in the US, Luciana et al., 2018; The Consortium on Vulnerability to Externalizing Disorders and Addictions Study in India, Zhang et al., 2020). Greater experiential diversity from adolescents comprising larger datasets will not only make this work more representative, it may also help parse contributions of cultural, social, and socioeconomic factors to adolescent neurodevelopment. A related goal will be for adolescent developmental neuroscience to continue making strides in following best practices in open research (Allen and Mehler, 2019) and validate results across discovery and replication subsamples or with larger, publicly available datasets (Simmons et al., 2021). While making the science more rigorous and broadening our sampling pool, we should also form working relationships with our participants, given that they are experts of their own experiences. For example, qualitative interviews with youth can be invaluable assets when developing culturally inclusive measures of emotion socialization or regulation strategy usage.

Secondly, context might be better incorporated into research on self-regulation neurodevelopment by crafting experimental paradigms that bolster generalizability and ecological validity. Many cognitive and emotion regulation paradigms currently use contrived computer tasks that do not reflect real-word scenarios, stressing the need for naturalistic approaches. Using more socially interactive paradigms that incorporate flexible neuroimaging methods (e.g., functional near-infrared spectroscopy; Pinti et al., 2020) and rich, naturalistic emotional stimuli (Sonkusare et al., 2019) will provide more robust evidence on the ways that adolescents self-regulate, especially in the presence of caregivers and peers. Future training paradigms should also incorporate neural measures of training efficacy to elucidate the ways continued practice drives corticolimbic adaptation. Relating behavioral outcomes to changes in neural function stands to inform our understanding of adolescent self-regulation development and could also help generate stronger training paradigms that benchmark neural outcomes.

The third and final recommendation for incorporating context into neuroscientific literature involves characterizing more complex, interwoven associations between environments and neurodevelopment. As Fig. 1 suggests, contextual factors do not exist in isolation. Interactive and combinatory effects of adolescents’ socioeconomic, interpersonal, and cultural environments on their regulatory neurodevelopment likely introduce additional variation in developmental outcomes. For example, Herd et al. (2020) demonstrated that adolescents experiencing greater socioeconomic disadvantage tend to report lower relationship quality with caregivers, which longitudinally tracks with slower development of emotion regulation skills and lower levels of situationally appropriate empathy and emotional self-awareness. This also suggests that aspects of the social environment in the home may buffer against emotion dysregulation for adolescents experiencing material disadvantage and could serve as a candidate intervention target. Moreover, constructs like SES are indirect measures of social and material capital, and it thus behooves researchers to think critically about which facets of SES inform their hypotheses, especially as it relates to larger sociopolitical contexts within the US and around the world. For example, socioeconomically disadvantaged adolescents in urban centers are more likely to identify as a racial or ethnic minority, experience greater pollution exposure with less access to greenspaces, and are at higher risk for developing emotional disorders (Dai, 2011, Rudolph et al., 2014, Wolch et al., 2014). The degree to which SES deleteriously impacts brain development also may depend on whether or not structural supports exist, such as state-level anti-poverty programs (Weissman et al., 2023). As such, these associations are embedded within a far more complex milieu of structural factors that extend beyond neighborhoods and thus require an intersectional lens when interpreting associations centered on disadvantage (Webb et al., 2022).

## Declaration of Competing Interest

The authors declare that they have no known competing financial interests or personal relationships that could have appeared to influence the work reported in this paper.

## Data Availability

No data was used for the research described in the article.

## Footnotes

1 The current section covers brain development in the context of circulating gonadal hormones and pubertal stage. As such, the framing of “female” and “male” human adolescents is an intentional one that short hands the medically assigned sex of children at birth. In subsequent sections, we also consider the experiences of transgender youth, whose sex assigned at birth (and presumed gender) does not reflect their gender identity.
